# Implementation of a rapid HIV testing program in psychiatric inpatient wards

**DOI:** 10.1186/1471-2334-14-S2-P6

**Published:** 2014-05-23

**Authors:** Maryann J Popiel, Vikram Duvvi, Albert Turkieh, Ethan Cowan, Yvette Calderon, Kelly M Chacón, Sara Rahman, John Y Rhee, Adam Kornmehl, Jason Leider

**Affiliations:** 1 Albert Einstein College of Medicine, Bronx, USA

## Aim

According to New York State law, HIV testing must be offered to every patient aged 13-64. However, acute mental health hospitalizations can be difficult settings in which to conduct HIV screening. Because individuals with mental illness have higher rates of HIV infection, routine testing of stabilized patients in psychiatric inpatient wards could be beneficial. This study describes the implementation of a rapid HIV testing program in this nontraditional setting.

## Methods

This prospective, descriptive study was conducted in a psychiatric inpatient ward over 22 days. A convenience sample of patients deemed to have capacity to consent by a team of physicians were recruited by Public Health Advocates (PHAs). PHAs collected demographic characteristics and risk factors during targeted counseling sessions. Chart reviews were conducted to assess psychiatric diagnoses and sexual/drug abuse history. Rates of test acceptances were tracked during the last 8 days of the study.

## Results

346 patients were tested for HIV. Demographic characteristics of the participants were: 60.1% male, 43.4% Hispanic, and 40.5% Black. Mean age was 37.7 ± 12.8. 36.7% do not engage in regular medical care. As per CDC criterion, 27.7% of participants were high-risk for acquiring HIV. Risk factors were: multiple sex partners (21.7%), injection drug use (5.8%), sex for commodities (4.9%), sex with an injection drug user (3.5%), or sex with an HIV-positive partner (1.5%). Psychiatric diagnoses were: Schizophrenic/Psychotic/Affective (64.5%), Depression (15.0%), and Bipolar (14.2%). Drug abuse was noted in 31.2% of cases, and 7.5% reported sexual abuse history. One patient was confirmed HIV positive and linked to outpatient HIV care. In last 8 days of study, 240 of 322 consentable patients were offered the HIV test (74.5%), of which 149 accepted (62.1%).

## Conclusion

A rapid HIV testing program with multidisciplinary staff buy-in can test a large number of patients in a psychiatric inpatient ward. Expansion of HIV screening initiatives to nontraditional settings can increase access to testing for high-risk populations that may not otherwise engage in primary care.

